# Molecular imaging of the pulmonary circulation in health and disease

**DOI:** 10.1007/s40336-014-0076-9

**Published:** 2014-09-09

**Authors:** Jocelyn Dupuis, François Harel, Quang T. Nguyen

**Affiliations:** 1 Research Center, Montreal Heart Institute, 5000 Belanger Street, Montreal, QC H1T 1C8 Canada; 2 Department of Medicine, Université de Montréal, Montreal, QC Canada; 3 Department of Radiology, Radio-Oncology and Nuclear Medicine Université de Montréal, Montreal, QC Canada

**Keywords:** Molecular imaging, Lung remodeling, Endothelium, Fibrosis, Apoptosis, Proliferation

## Abstract

The pulmonary circulation, at the unique crossroads between the left and the right heart, is submitted to large physiologic hemodynamic variations and possesses numerous important metabolic functions mediated through its vast endothelial surface. There are many pathologic conditions that can directly or indirectly affect the pulmonary vasculature and modify its physiology and functions. Pulmonary hypertension, the end result of many of these affections, is unfortunately diagnosed too late in the disease process, meaning that there is a crying need for earlier diagnosis and surrogate markers of disease progression and regression. By targeting endothelial, medial and adventitial targets of the pulmonary vasculature, novel molecular imaging agents could provide early detection of physiologic and biologic perturbation in the pulmonary circulation. This review provides the rationale for the development of molecular imaging agents for the diagnosis and follow-up of disorders of the pulmonary circulation and discusses promising targets for SPECT and positron emission tomographic imaging.

## Introduction

With the exception of labeled macroaggregates of albumin (MAA), used almost exclusively for the diagnosis of pulmonary embolism, there is currently no radionuclide agent routinely employed to study the pulmonary circulation in humans. Positioned at the unique crossroads between the right and the left heart, the pulmonary circulation is submitted to large physiologic hemodynamic variations serving gas exchanges. Furthermore, the pulmonary vascular endothelium represents a vast surface area responsible for numerous important metabolic functions affecting systemic functions. Many pathologic conditions directly or indirectly affect the pulmonary circulation and modify molecular pathways contained within the different layers of the pulmonary vasculature: the endothelium, media and adventitia. In this review, we discuss the opportunities and challenges involved in developing molecular imaging agents for detecting normal physiologic variations and pathologic disorders affecting the pulmonary circulation.

### Anatomy and physiology of the pulmonary circulation

The pulmonary circulation serves its primary function of gas exchange through a very large capillary surface area. By virtue of its great capacitance and low resistance, it is able to accommodate sizeable physiologic variations of the cardiac output with little variation in mean pulmonary artery pressure. This unique capacity is in part due to the postural perfusion gradient resulting from the gravity-dependent interplay between the alveolar pressure and the pulmonary capillary pressure in different regions of the lungs. Therefore gravity and other factors, to be detailed later, result in uneven spatial distribution of pulmonary perfusion.

With increases in pulmonary blood flow, such as during exercise, lung vascular recruitment occurs in the less dependent regions of the lungs leading to modification of the spatial distribution of blood flow. In diseases causing a progressive loss of pulmonary microcirculation, such as pulmonary hypertension (PH), the impact of this loss on the spatial distribution of pulmonary perfusion is currently uncertain. Therefore, this parameter is not clinically utilized to detect and monitor disease progression.

**Fig. 1:**
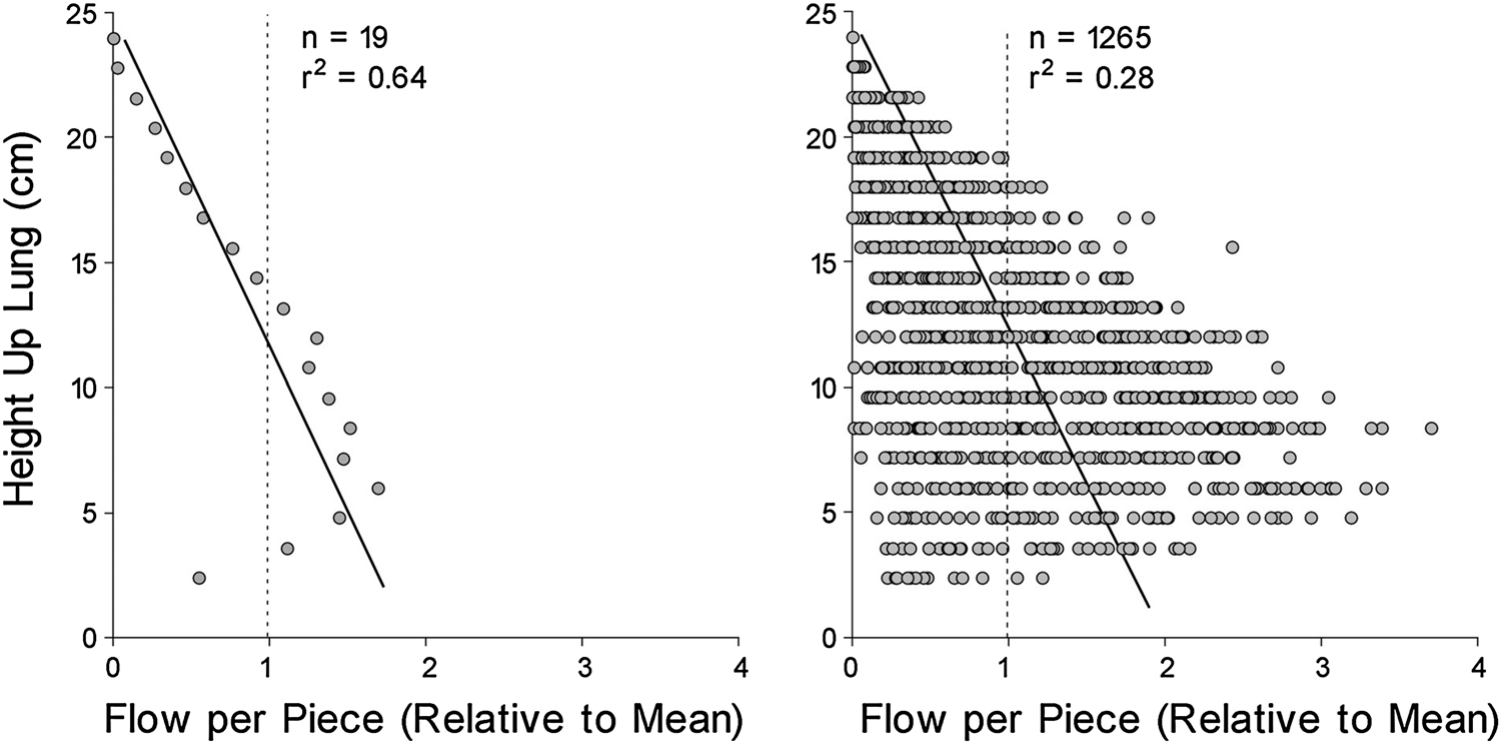
Blood flow as a function of height up the lung in an upright primate. Data are from 1,265 pieces of lung (2 cm^3^ in volume) and were obtained using the microsphere method. *Left* data averaged within horizontal planes to reproduce the spatial resolution available at the time the gravitational model was conceptualized. *Right* same data but at a resolution that permits the heterogeneity of perfusion to be observed. At the lower spatial resolution, the data are remarkably similar to those of the zone model described by Hughes and West [1–3] and gravity appears to be a major determinant of perfusion (*r* ^2^ = 0.640). However, at the higher resolution, gravity can account for at most 28 % of the variability in perfusion. Reproduced with permission from the Journal of Applied Physiology: Glenny [11]

The first studies describing the heterogeneity of lung perfusion led to the “zone model” of lung perfusion developed by West et al. [1, 2]. This classical physiologic model, still taught today, relies principally on the gravitational gradient to describe four zones of perfusion, in the upright posture, from the apex to the base of the lungs. This model, resulting from the interplay between arteriolar, venular and alveolar pressures, depicts increasing perfusion from the apex to the base of the lungs except in zone 4, where it was postulated that compression of extra-alveolar vessels results in decreasing perfusion [3] (Fig. 1). Since then, numerous studies have confirmed a gravitational lung perfusion gradient in upright, supine and prone positions in man as well as in quadrupeds [4–10]. Besides the effect of gravity on perfusion pressure, the so called “slinky” effect also contributes to greater perfusion in the most dependent regions of the lungs as gravity causes tissue compression and greater lung parenchymal density [5].

However, factors other than gravity also contribute to the heterogeneity of lung perfusion, and the question of whether gravity is the principal determinant of the spatial distribution of lung perfusion has been the subject of much heated debate [11–15]. There is indeed evidence of perfusion heterogeneity even in isogravitational planes. Hakim et al. [4, 16] demonstrated a decreasing centro-peripheral perfusion gradient in isogravitational planes of human and canine lungs and hypothesized that this observation resulted from varying regional vascular conductance at branching points. Indeed, studies performed in microgravity environments revealed that some lung perfusion heterogeneity persisted [17]. A novel fractal model incorporating isogravitational heterogeneity was proposed by Glenny et al. [18–20], who suggested that as the spatial resolution of the instruments of measure is improved, isogravitational perfusion heterogeneity is revealed [11] (Fig. 1).

Although the relative importance of the determinants of the spatial distribution of pulmonary perfusion is the subject of an ongoing debate, there is consensus on its ultimate finality. The heterogeneity of lung perfusion confers great capacitance on the pulmonary vasculature and the possibility to increase (recruit) tissue perfusion for gas exchanges in response to increasing cardiac output. Using the multiple indicator-dilution technique in exercising dogs, we demonstrated that the metabolically active pulmonary vascular surface area increased almost linearly with tripling of blood flow [21–23]. Furthermore, lung vascular recruitment continues to occur even after full lung tissue recruitment measured from the tracer-accessible extravascular lung water [21–23]. There is therefore a pulmonary vascular “reserve” that can accommodate the increase in cardiac output and expand the surface available for gas exchanges and pulmonary metabolic functions. Lung vascular recruitment in response to increasing blood flow will accordingly modify the spatial distribution of pulmonary perfusion with a reduction in the gravitational gradient component [9, 10, 24]. Study of the spatial distribution of the metabolically active pulmonary circulation at rest and with increasing pulmonary blood flow, such as exercise, could therefore provide a unique insight into the capacity of the lung to recruit vascular surface area. More importantly, study of the spatial distribution of perfusion in conditions associated with a loss of recruitable pulmonary perfusion, such as PH, could provide a unique method for detecting disease earlier than is currently possible.

## The difficulty and importance of early detection of pulmonary vascular disease

Pulmonary hypertension (PH) results from various clinical conditions and is defined as a mean pulmonary artery pressure ≥25 mmHg at rest. The pathophysiology-based classification of PH comprises five groups [25] (Table 1). Although not the most prevalent form, Group 1 PH is attracting increasing attention, with novel selective pharmacologic therapies having been developed and approved in the past 10 years. Group 1 PH, often referred to as pulmonary arterial hypertension (PAH), is a severe angioproliferative disease of the pulmonary microcirculation which causes progressive obliteration of distal pulmonary arteries measuring less than 500 µm. It may be idiopathic, hereditary or linked to various disorders such as collagen vascular diseases, portal hypertension, congenital heart disease, HIV infection, drugs and toxins, schistosomiasis and others [25].

**Table 1:** Classification of pulmonary hypertension

1.Pulmonary arterial hypertension
1.1.Idiopathic PAH
1.2.Heritable PAH
1.2.1.BMPR2 mutations
1.2.2.ALK-1, ENG, SMAD9, CAV1, KCNK3 mutations
1.2.3.Unknown
1.3.Drug and toxin induced
1.4.Associated with
1.4.1.Connective tissue disease
1.4.2.HIV infection
1.4.3.Portal hypertension
1.4.4.Congenital Heart Disease
1.4.5.Schistosomiasis
1′ Pulmonary veno-occlusive disease and/or pulmonary capillary hemangiomatosis
1″ Persistent pulmonary hypertension of the newborn (PPHN)
2.Pulmonary hypertension due to left heart disease
2.1.Left ventricular systolic dysfunction
2.2.Left ventricular diastolic dysfunction
2.3.Valvular disease
2.4.Congenital/acquired left heart inflow/outflow tract obstruction and congenital cardiomyopathies
3.Pulmonary hypertension due to lung diseases and/or hypoxia
3.1.Chronic obstructive pulmonary disease
3.2.Interstitial lung disease
3.3.Other pulmonary diseases with mixed restrictive and obstructive pattern
3.4.Sleep-disordered breathing
3.5.Alveolar hypoventilation disorders
3.6.Chronic exposure to high altitude
3.7.Developmental lung diseases
4.Chronic thromboembolic pulmonary hypertension (CTEPH)
5.Pulmonary hypertension with unclear multifactorial mechanisms

Classification from the proceedings of the 5th World Symposium on PH [100]

Despite modern diagnostic modalities, more than 2 years will typically elapse between initial medical contact and the diagnosis of group 1 PH [26–28]. At the time of diagnosis, up to 75 % of subjects are already in NYHA functional class III or IV [26]. Although specific PAH therapies are available, the prognosis of this condition remains exceedingly poor. In the REVEAL study registry, the one-, three-, five- and seven-year survival rates from time of diagnosis were 85, 68, 57 and 49 %, respectively, in patients with all-cause PAH [29]. For patients with idiopathic PAH, heritable PAH or drug-induced PAH, the three-year survival was 58.2 %. There is clearly an urgent need for earlier detection of pulmonary vascular disease [30, 31].

Other groups of PH, such as PH due to left heart disease (group 2) and PH due to lung parenchymal disease (group 3) are much more prevalent and confer a very poor prognosis in affected subjects. Unfortunately, there are no specific therapies currently approved for these groups of PH and evidence-based guidelines for a diagnostic and monitoring imaging approach are lacking.

The main reason for the delay in the diagnosis of PH resides in the recruitable pulmonary vascular reserve: it is estimated that more than 50 % of the pulmonary vascular bed is obliterated before there is a detectable rise in mean resting pulmonary artery pressure [30]. Indeed, normal subjects can withstand a unilateral pneumonectomy without significant hemodynamic PH in the remaining lung [32, 33]. Some advocate the measurement of pulmonary artery pressure at exercise to allow earlier detection of PH. Although pulmonary artery systolic pressure can be non-invasively measured during exercise with echocardiography, the approach is currently debated due to the lack of a specific upper-bound estimate and is not recommended as a screening test for PH [34, 35]. Because of the recruitable vascular reserve, the pressure rise in PH is a late event. Thus, there is a need for methods allowing earlier direct assessment the status of the pulmonary circulation. As novel pharmacologic agents continue to be developed, earlier diagnosis and therapy may have greater benefits on morbidity and mortality. Evaluation of the spatial distribution of pulmonary perfusion at rest and during exercise may provide an earlier and sensitive insight into the status of the pulmonary circulation. Modification of the spatial distribution of lung perfusion may be detectable well before a significant rise in resting pulmonary artery pressure.

Molecular imaging agents targeting the pulmonary circulation therefore hold promise as sensitive early indicators of pulmonary vascular disease and of its progression or regression. Currently, the diagnosis of PH (Table 1) requires an invasive right heart catheterization and there is no consensus on what non-invasive clinical parameter is the best surrogate marker of severity and prognosis.

## Metabolic functions of the pulmonary vascular endothelium

**Fig. 2:**
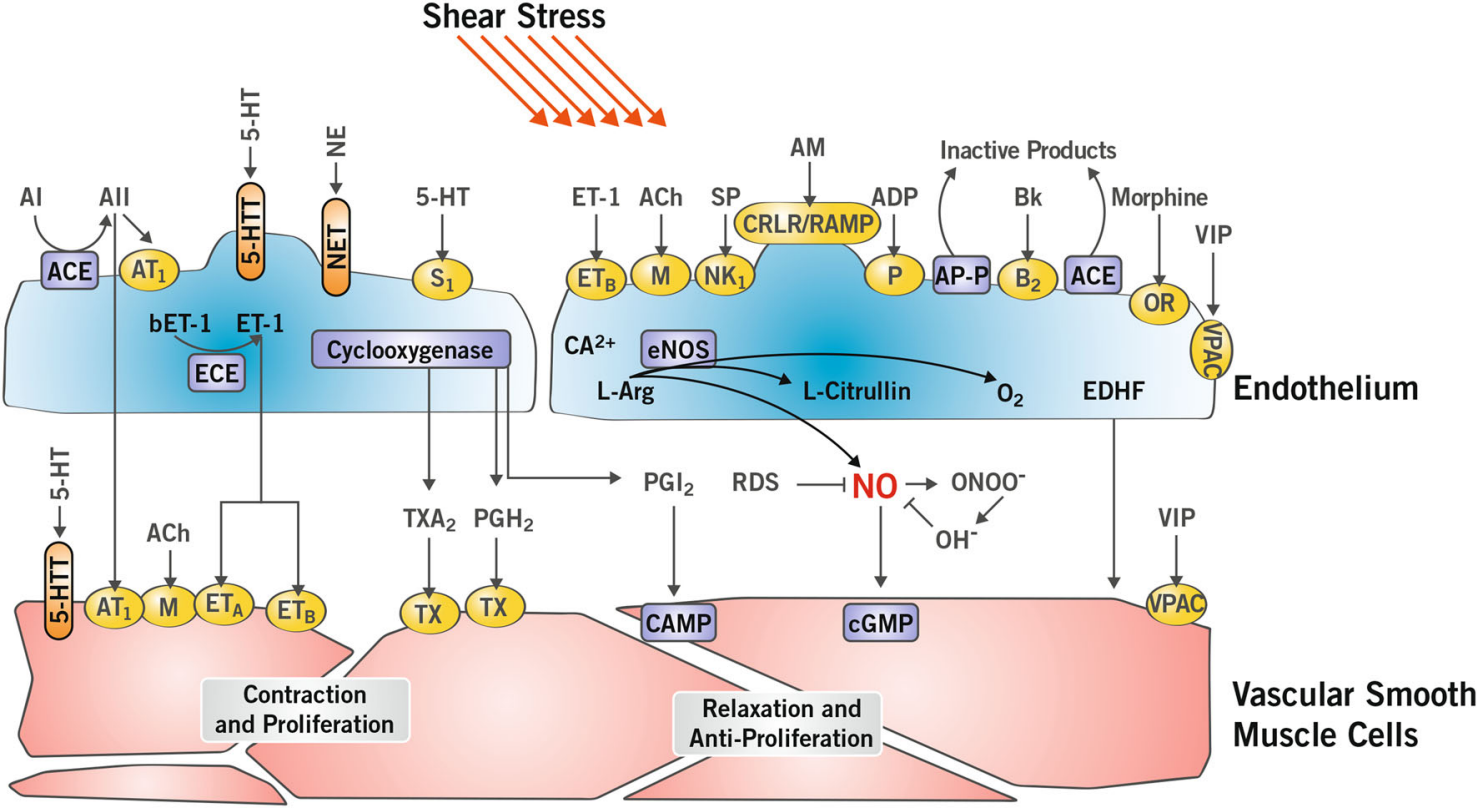
Biologic functions of the endothelial and muscular layers of the pulmonary vasculature. Various mediators are produced, transformed or inactivated by the pulmonary vascular endothelial cells. Specific receptors and transporters are expressed by endothelial and vascular smooth muscle cells. Nitric oxide (NO); endothelium-derived hyperpolarizing factor (EDHF); prostacyclin (PGI_2_); angiotensin-converting enzyme (ACE); acetylcholine (Ach); angiotensin I (AI); angiotensin II (AII); angiotensin 1 receptor (AT1); bradykinin (Bk); cyclo-oxygenase (COX); endothelin-converting enzyme (ECE); endothelin A and B receptors (ET_A_, ET_B_); endothelin-1 (ET-1); l-arginine (L-Arg); prostaglandin H2 (PGH2); reactive oxygen species (ROS); serotoninergic receptor (S1); thromboxane receptor (TH); thrombin (Thr); thromboxane A2 (TXA2); serotonin (5-HT); opioid receptor (OR); calcitonin receptor-like receptor-receptor activity modifying protein (CRLR-RAMP); adrenomedullin (AM); neurokinin receptor (NK-1); substance P (SP); vasoactive intestinal peptide receptor (VPAC); vasoactive intestinal peptide (VIP); aminopeptidase P (AP-P); norepinephrine transporter (NET); norepinephrine (NE); serotonin transporter (5-HTT) (color figure online)

The pulmonary circulation has numerous important metabolic functions exerted through its specialized capillary endothelial cells [36]: it produces, activates, modifies and degrades circulating mediators affecting not only the underlying smooth muscle cells but also the systemic circulation and organs. Endothelial functions of potential interest for molecular imaging are depicted in Fig. 2. Endothelial dysfunction is an early event in pulmonary vascular disease and many endothelial functions are potential targets for molecular imaging. Prototypical targets include the angiotensin-converting enzyme (ACE), serotonin (5-HT) and norepinephrine (NE) transporters, endothelin production and clearance, and finally the production of the endothelium-derived relaxing factor nitric oxide (NO). ACE is an ectoenzyme located at the luminal surface of the pulmonary vascular endothelium, responsible for converting angiotensin I into angiotensin II, a potent systemic vasoconstrictor. The pulmonary circulation is the major site of ACE activity and radiolabeled ACE substrates have been used to measure lung capillary recruitment with increasing pulmonary blood flow [22, 37]. By contrast, serotonin and NE are removed from circulation by the lungs [38]. Lung uptake of ^14^C-serotonin and ^3^H-NE have been used to demonstrate lung vascular recruitment and the increase in metabolically active pulmonary vascular surface with exercise [21, 23]. The pulmonary vascular endothelium is an important site of production and clearance of circulating endothelin 1 (ET-1), a potent vasoconstrictor and proliferator implicated in the pathophysiology of PH. Production of both endothelial prostacycline (PGI_2_) and NO is reduced in PAH and related to increased vascular tone and an increased proliferative profile of the endothelium and vascular smooth muscle cells.

It is generally recognized that endothelial dysfunction is an early initiating event in the development of group I PH and contributes to the abnormal pulmonary vascular reactivity and remodeling of all PH groups. Endothelial metabolic functions are therefore putative molecular imaging targets for monitoring disorders affecting the pulmonary circulation. Furthermore, increased endothelial cell apoptosis associated with quasi-clonal proliferation of activated apoptotic-resistant endothelial cells is a hallmark of PAH, which is characterized by activation of these respective biologic pathways [39].

## Molecular imaging of the pulmonary vasculature

Many molecular imaging agents have demonstrated lung uptake and potential utility in the diagnosis of pulmonary vascular disease. Table 2 lists some of these agents and their molecular targets. However, very few have been tested in human subjects. This is by no means an exhaustive list as agents developed for other purposes may also allow pulmonary vascular imaging, even though they were not specifically evaluated for this purpose. We here review and discuss in greater detail some agents of interest that have more specifically been tested as agents for the evaluation of PH.

**Table 2:** Molecular PET and SPECT radioligands used to image the pulmonary vasculature

Target	Radioligand
Opioid receptor	^3^H-fentanyl [101], ^11^C-MeJDTic [102]
Adrenomedullin receptor	^99m^Tc-AM-L [53], ^99m^Tc-PulmoBind [51]
Beta-adrenoreceptor	^3^H-propranolol [103], ^14^C-propranolol [104, 105]
Serotonin receptor	^11^C-GSK215083 [106]
Dopamine receptor	^11^C-NNC 112 [107]
Endothelin ET(B) receptor	^18^F-BQ3020 [64]
Neurokinin NK-1 receptor	^18^F-SPA-RQ [108]
Vasoactive intestinal peptide receptor	^123^I -VIP [109, 110]
Glucagon-like peptide-1 receptor	[Lys(40)(Ahx-DOTA-(68)Ga)NH(2)]-exendin-4 [111, 112]
Norepinephrine transporter	^123^I-MIBG [70–72, 113–115]
Serotonin transporter	^123^I-ADAM [116, 117], ^11^C-DASB [118], ^14^C-serotonin [103],
	^123^I-FP-CIT [119, 120]
	^123^I-iodoamphetamine [121], ^123^I-HIPDM [122]
Glucose transporter	^18^F-FDG [79–81]
MMPs, gelatinase	^99m^Tc-DTPA-CLP [123], ^99m^Tc-CTT [124]
Aminopeptidase P	^125^I-833c [125], ^99m^Tc-mAPP [126]
Externalized phosphatidylserine	^99m^Tc-annexin V [76–78]
Angiotensin-converting enzyme	^3^H-BPAP [65, 66], ^11^C-zofenoprilat [127], ^18^F-fluorocaptopril [68], ^99m^Tc-lisinopril [128]

^11^C-MeJDTic = ^11^C–N-methylated derivative of JDTic ((3*R*)-7-hydroxy-*N*-[(2*S*)-1-[(3*R*,4*R*)-4-(3-hydroxyphenyl)-3,4-dimethylpiperidin-1-yl]-3-methylbutan-2-yl]-1,2,3,4-tetrahydroisoquinoline-3-carboxamide); ^11^C-NNC 112 = ^11^C((+)-8-chloro-5-(7-benzofuranyl)-7-hydroxy-3-methyl-2,3,4,5-tetrahydro-1*H*-3-benzazepine); ^18^F-BQ3020 = ^18^F-([Ala^11,15^]Ac-ET-1(6–21)); ^18^F-SPA-RQ = ^18^F-[2-fluoromethoxy-5-(5-trifluoromethyl-tetrazol-1-yl)-benzyl]-[(2S,3S)-2-phenyl-piperidin-3-yl)amine]; ^123^I-MIBG = ^123^I-metaiodobenzyl guanidine; ^123^I-ADAM = ^123^I-2-((2-((dimethylamino)methyl) phenyl)thio)-5-iodophenylamine; ^11^C-DASB = ^11^C-3-amino-4-(2-dimethylaminomethyl-phenylsulfanyl) benzonitrile; ^123^I-FP-CIT: ^123^I-N-ω-fluoropropyl-2β-carbomethoxy-3β-(4-iodophenyl) nortropane; ^123^I-iodoamphetamine = ^123^I-*N*-isopropyl p-iodoamphetamine; ^123^I-HIPDM = ^123^I-*N,N,N*′-trimethyl-N-(2-hydroxy-3-methyl-5 iodobenzyl)-1, 3 propanediamine; ^18^F-FDG: ^18^F-fluoro-2-deoxy-2-d-glucose; ^99m^Tc-DTPA-CLP = ^99m^Tc-DTPA-Cys-Leu-Pro-Gly-His-Trp-Gly-Phe-Pro-Ser-Cys; ^99m^Tc-CTT = ^99m^Tc-Cys-Thr-Thr-His-Trp-Gly-Phe-Thr-Leu-Cys; ^125^I-833c = ^125^I- radiolabeled aminopeptidase P-specific recombinant antibody; ^99m^Tc-mAPP = ^99m^Tc-radiolabeled monoclonal antibody to aminopeptidase; ^3^H-BPAP = ^3^H-benzoyl-phenylalanyl-alanyl-proline; ^11^C-zofenoprilat = ^11^C-(4*S*)-1-[(*S*)-3-Mercapto-2-methylpropanoyl]-4-phenylthio-l-proline. AM-L = linear form of human adrenomedullin

### Endothelium

Various molecular tracers targeting the pulmonary vascular endothelium have been tested in pre-clinical studies although few have been evaluated in humans. Here we discuss four endothelial metabolic functions that have been explored in some detail for their potential clinical relevance: the adrenomedullin (AM) receptor, the endothelin-B receptor (ET_B_), ACE and the NE transporter.

**Fig. 3:**
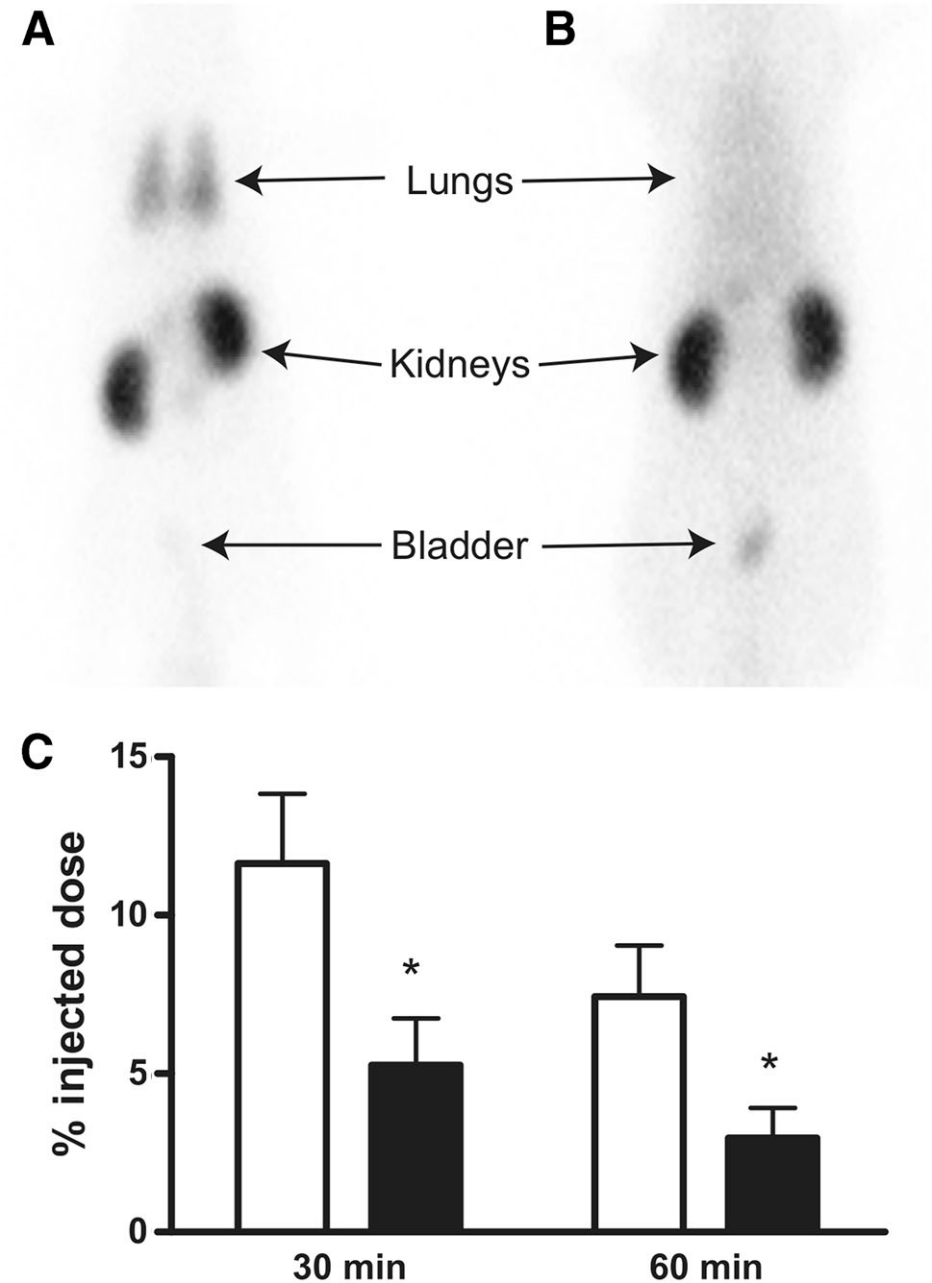
Molecular imaging of pulmonary arterial hypertension using an adrenomedullin receptor ligand. Images obtained 30 min after an i.v. injection of ^99m^Tc-PulmoBind in **a** vehicle-treated Sprague–Dawley rat and **b** monocrotaline-treated rat (pulmonary arterial hypertension model). **c** Static evaluations of the presence of the radiotracer in lungs 30 and 60 min after injection. *p < 0.05 for vehicle-treated rats (*white bars*) versus monocrotaline-treated rats (*black bars*). This research was originally published in the *Journal of Nuclear Medicine*: Letourneau et al., PulmoBind an adrenomedullin-based molecular lung imaging tool, 2013; vol. 54, 1789–1796. © by the Society of Nuclear Medicine and Molecular Imaging, Inc. [51]

Human AM, a member of the calcitonin gene-related peptide family, is a 52-amino-acid multifunctional regulatory peptide expressed in a wide range of tissues but mainly in the adrenal medulla, ventricle, kidneys and lungs [40–42]. Its specific heterodimeric receptor is composed of the calcitonin-like receptor and the receptor activity-modifying protein 2 or 3 [43]. The AM receptor is abundantly expressed in human alveolar capillaries and mostly distributed at the luminal surface of the vascular endothelium [44–47]. Accordingly, the lungs contain specific AM-binding sites at a density higher than any other organ studied [47]. We have previously established that the lungs are a primary site for plasma AM clearance and therefore modulate its circulating levels [48]. In fact, the AM receptor acts as a clearance receptor in the pulmonary vascular bed [49]. On the basis of this evidence, we hypothesized that radiolabeled AM derivatives could be used as non-invasive imaging tracers to evaluate the integrity of the pulmonary circulation. Through rational design and structure–activity relationship studies we developed various AM derivatives [50] able to maintain binding affinity with the specific receptor without significant biologic effects at the lung scan dose, while enabling the addition of a chelating moiety for a suitable radioisotope [51]. These derivatives allowed good quality lung imaging enabling the detection of large pulmonary perfusion defects mimicking pulmonary embolism [52], but also microcirculatory pulmonary occlusion in the monocrotaline model of PAH [53] (Fig. 3). A lead compound possessing the desired properties was selected [51] and called PulmoBind. In pre-clinical studies, ^99m^Tc-PulmoBind displays all the qualities desired of a molecular imaging agent in nuclear medicine: sizeable first pass and prolonged uptake by the lungs and quick plasma clearance with elimination by both the liver and kidneys [51]. A human phase I study of PulmoBind in normal human subjects was recently completed (Clinicaltrials.gov NCT01539889) [54]. We demonstrated that PulmoBind is safe while providing superior quality lung imaging compared to ^99m^Tc-MAA. A phase II study of safety and proof of concept in subjects with PAH is in the planning phase. We hypothesize that quantitative total lung uptake and kinetic parameters after PulmoBind injection will be of value in the evaluation of all the PH groups (Table 1). Furthermore, evaluation of the spatial distribution of PulmoBind uptake may provide a unique insight into modifications of the metabolically active pulmonary vascular surface area in both physiology and pathology.

Endothelin-1 (ET-1) is a potent vasoconstrictor and proliferator peptide produced by the vascular endothelium. The ET system is activated in and contributes to all groups of PH. ET exerts its biologic effects mainly by acting on two receptor sub-types: ET_A_ and ET_B_ [55, 56]. While ET_A_ is expressed only on smooth muscle cells, ET_B_ is expressed both on smooth muscle cells and on the endothelium [57]. The endothelial ET_B_ is densely expressed in the pulmonary circulation that acts as a clearance site for circulating ET-1 [58]. Using the indicator-dilution technique in man, approximately 47 % of circulating ET-1 is cleared by the lungs during a single circulatory transit time [59, 60]. Furthermore, clearance seemed to be differently affected by the various PH groups: it was reduced in group 2 PH but surprisingly preserved in some subjects with group 1 PH and chronic thromboembolic PH (group 4) [61]. Pulmonary clearance of ET-1 was reduced in patients with systolic heart failure (group 2 PH) in relation to the severity of PH [62]. In vivo imaging of the pulmonary endothelial ET_B_ receptor was performed by Davenport using ^18^F-ET-1 and the radiolabeled selective ET_B_ antagonist ^18^F-BQ-3020 [63, 64]. In animal studies, using a micro-PET, these tracers were rapidly and substantially taken up by the pulmonary circulation, resulting in good lung imaging. The specific role of the endothelial ET_B_ was confirmed by the inhibition of lung uptake after the administration of another selective ET_B_ antagonist. Unfortunately no further explorations with these tracers have been performed in pulmonary vascular disorders or in human subjects.

Angiotensin-converting enzyme (ACE) is an ectoenzyme located at the luminal surface of the pulmonary vascular endothelium and it is responsible for the hydrolysis of angiotensin I into the potent vasoconstrictor/proliferator angiotensin II (Ang-II). The lungs are the main site of circulating Ang-II production. Various ACE substrates and antagonists have been developed and labeled to study pulmonary ACE activity in vivo. Using the indicator-dilution approach, ACE substrates have been validated as tools for studying the metabolically active pulmonary vascular surface area in animals [22, 37] and in humans [65]. Pulmonary endothelial ACE activity is reduced by acute vascular lung injury and in subjects with PH [66, 67]. ACE inhibitors are among the most widely prescribed cardiovascular drugs and molecular lung imaging has been performed in humans using ^18^F-fluorocaptopril and ^18^F-lisinopril. Lung ACE imaging was performed in human subjects with ^18^F-fluorocaptopril and a three-compartment model was used to estimate total ACE binding [68]. The authors found marked reduction of ACE binding in subjects with PAH and could image and quantify the pharmacologic efficacy of ACE antagonists.

The lungs extract circulating NE by a specific transporter located in the vascular endothelium and this biologic function has been exploited for molecular imaging of the pulmonary vasculature. This energy-dependent process was imaged using ^123^I-metaiodobenzylguanidine (MIBG) scintigraphy, studying the lung uptake and wash-out of the radioimaging agent [69]. Studies have shown diminished lung extraction of ^123^I-MIBG in patients with chronic obstructive pulmonary disease, pulmonary fibrosis, vasculitis, and after radiotherapy and high altitude hypoxia [70–74]. Despite its potential, this agent is not currently used for diagnosis of lung vascular disorders. The lung imaging technique with ^123^I-MIBG has never been standardized and the biology of the tracer remains uncertain as lung neuronal uptake, in addition to endothelial uptake, likely contributes to the kinetics of this tracer [69].

### Apoptosis and proliferation

The pathophysiology of PH is complex and incompletely understood. It comprises early endothelial injury (dysfunction) with inflammation and dysregulation of growth factors [75]. This translates into endothelial apoptosis followed by proliferation of apoptosis-resistant endothelial cells and of vascular smooth muscle cells, leading to the angioproliferative phenotype pathognomonic of group 1 PH (PAH) with the formation of plexiform lesions. Eventually, all types of PH will display pulmonary artery smooth muscle cell proliferation and fibroblast proliferation with possible obliteration of the vascular lumen.

Annexin V is a ubiquitous protein that binds to phosphatidylserine expressed on the surface of apoptotic cells but also on “stressed” or injured cells [76]. Radiolabeled annexin V has been used as a cancer imaging agent but also has potential for a variety of pulmonary pathologies involving apoptosis and inflammation [76], including PH. In the monocrotaline model of PAH, ^99m^Tc-annexin V imaging showed clear increases in pulmonary apoptosis, which regressed with effective therapy [77]. In a murine model of acute lung transplant rejection, ^99m^Tc-annexin V lung uptake was increased in relation to the severity of histological rejection [78].

The metabolic glycolytic shift of proliferating cells can be detected using ^18^F-fluorodeoxy-glucose positron emission tomography (^18^F-FDG PET), a technique that is widely used in oncology. The paradigm of quasi-clonal proliferation of endothelial cells and vascular smooth muscle cell proliferation associated with PAH was tested in animal models and in human studies. In the murine monocrotaline model as well as in the hypoxia-Sugen model of PAH there is increased pulmonary ^18^F-FDG PET uptake, which occurs early and correlates with disease severity [79]. Furthermore, it is associated with increased expression of the GLUT1 transporter in both endothelial cells and pulmonary vascular smooth muscle cells. Lack of upregulation of the glucose transporter in inflammatory cells would suggest, at least in these models, that ^18^F-FDG PET can be used to monitor the vascular proliferative component of PAH [79]. In human PAH, two small studies revealed a mean increase of lung parenchymal ^18^F-FDG PET activity [80, 81]. The ^18^F-FDG PET activity was, however, heterogeneously distributed within the lungs and showed wide variability between subjects, with some patients having normal uptake. Although the within-lung heterogeneity would seem to be consistent with the known heterogeneity of histological pathology in PAH [75], the between-subject variability raises the concern that the ^18^F-FDG tracer may not gain access to some diseased areas due to blockade of pulmonary vessels, a problem inherent to a disease causing a reduction in perfusion. Indeed, and contrary to cancer in which proliferating tumors are generally highly vascularized, PH causes a reduction in perfusion that is proportional to the disease severity. Another limitation of ^18^F-FDG PET is its lack of specificity for the lung vasculature. Indeed, other lung conditions such as pulmonary fibrosis and diseases causing parenchymal lung damage are associated with increased ^18^F-FDG PET activity in regions of fibrosis [82].

### Agents used to measure pulmonary perfusion

Both particulate and non-particulate agents have been used to measure pulmonary perfusion. Although some are not molecular imaging agents per se, they are nevertheless here reviewed briefly.

Non-particulate agents that cross the alveolar blood-gas barrier may be used to measure pulmonary perfusion. Initially, radiolabeled carbon dioxide (C^15^O_2_) was used by inhalation in combination with detection systems using external scintigraphic probes. This allowed approximate measurement of regional pulmonary perfusion [1, 2, 83]. ^133^Xenon, previously used to quantify cerebral perfusion, has also been evaluated for pulmonary applications after first being dissolved in physiologic saline for intravenous injection [84, 85]. The introduction of planar detection systems (Anger Camera) has enabled the creation of images showing the distribution of pulmonary perfusion.

The method most frequently used for the measurement of pulmonary perfusion involves the injection of radiolabeled particles that lodge in the pulmonary vasculature through microembolization [86]. Depending on their size, these particles lodge in the smallest vessels they can reach. The MAA constitute the prototype of those substances [87–89]. These radiopharmaceuticals were found to be well suited to new technological developments in detection systems. Indeed, the addition of a tomographic camera (SPECT) has allowed the creation of three-dimensional images. The longer time required to obtain these images from the SPECT camera precluded the use of non-particulate radiopharmaceuticals. Therefore, MAA were used after labeling with ^99m^technetium or ^113m^Indium [90, 91]. Many studies have been conducted using this tracer and the ^99m^Tc-MAA are still widely used in clinical applications. Some groups have also used ^81m^krypton [92]. However, this radioisotope, with a very short half-life (13 s), required continuous infusion in order to create scintigraphic images. Although more accurate, the SPECT technique does not allow absolute quantitative analysis.

The introduction of the positron emission tomographic camera (PET scanner) as an absolute measuring system has driven the development and use of new radiopharmaceuticals. This camera includes an efficient attenuation correction system and is able to provide quantitative data on the biodistribution of radiotracers injected into patients. These tracers must be positron emitters. Of these, some, such as ^13^N_2_, have been used to measure pulmonary perfusion. ^13^N_2_ is not very soluble and when dissolved in saline, and after intravenous injection, it rapidly diffuses into the alveolar space during the lung’s first-pass transit [93–96]. The tracer travels preferentially into the active alveoli, i.e. alveoli perfused by functional pulmonary capillaries. Thus, the tracer uptake in the lungs is a real marker of pulmonary perfusion. Oxygen-15 (H_2_^15^O) has also been used [97–99]. Again, this very short-life radiotracer must be injected according to a constant infusion model. Moreover, the biodistribution of the tracer needs to be measured and these calculations require the use of compartmental analysis. Finally, positron-emitting particulate tracers have been developed for the quantification of pulmonary perfusion, including ^68^Ga-labeled MAA.

## Conclusions

There are numerous clinical disorders that can directly or indirectly impact the pulmonary circulation leading to the development of PH. Unfortunately, there is currently no non-invasive diagnostic modality that can provide direct information on the pathophysiologic processes affecting the different layers of the pulmonary vasculature: the endothelium, the media and the adventitia. Molecular imaging using innovative SPECT and PET radiotracers offers promising prospects for the evaluation of the pulmonary circulation in physiologic and pathologic conditions. More importantly, molecular imaging could provide earlier detection of the disease, prior to the development of overt hemodynamic PH. Endothelial dysfunction is an early event in the development of PH and, in this regard, the authors believe the most promising agents will target endothelial metabolic properties. Some promising studies have been performed with agents targeting the endothelin receptors, the ACE, the NE transporter and more recently the AM receptor. The latter is to be tested in an upcoming phase II study. The future of molecular imaging of the pulmonary circulation in health and disease is promising. We predict that multimodality imaging with SPECT/CT and PET/MRI will provide combined anatomical and functional information essential to diagnosis, follow-up and therapeutic decisions in this field.
